# Effects of Transcranial Direct Current Stimulation on Psychophysiological Responses to Maximal Incremental Exercise Test in Recreational Endurance Runners

**DOI:** 10.3389/fpsyg.2018.01867

**Published:** 2018-10-09

**Authors:** Carlo Baldari, Cosme F. Buzzachera, Marcelo Vitor-Costa, Juliano M. Gabardo, Andrea G. Bernardes, Leandro R. Altimari, Laura Guidetti

**Affiliations:** ^1^eCampus University, Novedrate, Italy; ^2^Department of Physical Education, North University of Paraná, Londrina, Brazil; ^3^Department of Physical Education, State University of Londrina, Londrina, Brazil; ^4^Department of Movement, Human and Health Sciences, University of Rome “Foro Italico”, Rome, Italy

**Keywords:** endurance performance, running, perception of effort, affective valence, tDCS

## Abstract

Previous studies have suggested that transcranial direct current stimulation (tDCS) might improve exercise performance and alter psychophysiological responses to exercise. However, it is presently unknown whether this simple technique has similar (or greater) effects on running performance. The purpose of this study was, therefore, to test the hypothesis that, compared with sham and cathodal tDCS, anodal tDCS applied over the M1 region would attenuate perception of effort, improve affective valence, and enhance exercise tolerance, regardless of changes in physiological responses, during maximal incremental exercise. In a double-blind, randomized, counterbalanced design, 13 healthy recreational endurance runners, aged 20–42 years, volunteered to participate in this study. On three separate occasions, the subjects performed an incremental ramp exercise test from rest to volitional exhaustion on a motor-driven treadmill following 20-min of brain stimulation with either placebo tDCS (sham) or real tDCS (cathodal and anodal). Breath-by-breath pulmonary gas exchange and ventilation and indices of muscle hemodynamics and oxygenation were collected continuously during the ramp exercise test. Ratings of perceived exertion (RPE) and affective valence in response to the ramp exercise test were also measured. Compared with sham, neither anodal tDCS nor cathodal tDCS altered the physiological responses to exercise (*P* > 0.05). Similarly, RPE and affective responses during the incremental ramp exercise test did not differ between the three experimental conditions at any time (*P* > 0.05). The exercise tolerance was also not significantly different following brain stimulation with either sham (533 ± 46 s) or real tDCS (anodal tDCS: 530 ± 44 s, and cathodal tDCS: 537 ± 40 s; *P* > 0.05). These results demonstrate that acute tDCS applied over the M1 region did not alter physiological responses, perceived exertion, affective valence, or exercise performance in recreational endurance runners.

## Introduction

Transcranial direct current stimulation (tDCS) has emerged as a minimally invasive form of cortical stimulation in which constant, weak electrical current passes through brain tissue via two or more small electrodes placed on the participant’s scalp for up to 20 min (Nitsche et al., 2008; Filmer et al., 2014). Recent research has shown that this simple technique can produce pronounced changes in cortical excitability (Bindman et al., 1964a,b; Nitsche and Paulus, 2000). The neuromodulatory effects of tDCS, however, depend on the polarity applied – anodal stimulation enhances, and cathodal stimulation diminishes, cortical excitability (Bindman et al., 1962; Nitsche et al., 2003). The reasons for these polarity-specific effects are complex and not entirely understood. Previous research has shown that anodal stimulation causes neuronal depolarization and increases cerebral excitability while cathodal stimulation has opposite effects (Bindman et al., 1962; Purpura and McMurtry, 1965). Although much remains unknown, there is a growing evidence that tDCS offers a promising approach for exploring brain-behavior relationships across a variety of cognitive, affective, sensory, perceptual, and motor domains (Filmer et al., 2014). To date, tDCS has been extensively used in human cognitive neuroscience to understand brain function and brain plasticity (Santarnecchi et al., 2015) and in the treatment of a variety of psychiatric and neurological disorders (Murphy et al., 2009; Kuo et al., 2014; Thibaut et al., 2014).

In recent years, there has been considerable interest in the utility of tDCS as a potential ergogenic aid to exercise performance (Angius et al., 2017). Several investigations have examined the acute effects of tDCS of the primary motor cortex (M1) on isometric time to task failure tests of isolated muscle groups, however, the literature is inconclusive; some authors have demonstrated improvements (Williams et al., 2013; Abdelmoula et al., 2016; Angius et al., 2016), whereas others show no effect on time to task failure of a sustained submaximal contraction (Kan et al., 2013; Muthalib et al., 2013). Similar inconsistencies in exercise performance have also been noted in continuous, dynamic, whole-body exercise studies (Angius et al., 2015; Okano et al., 2015; Vitor-Costa et al., 2015; Barwood et al., 2016). Some of the discrepancies between studies may be attributed to differences in electrode montage and location (Angius et al., 2017). Four recent studies have used different approaches to address this question and have produced somewhat disparate results. Okano et al. (2015) reported significantly improved peak power and reduced perception of effort following a cephalic tDCS montage with the anode over T3 of the insular cortex and with the cathode over the contralateral supraorbital area, fp2, on trained cyclists. These promising findings were not replicated in two separate studies using a similar tDCS stimulation paradigm (Barwood et al., 2016). Alternatively, Vitor-Costa et al. (2015) reported a significant improvement in time to exhaustion during cycling exercise following anodal tDCS applied over the M1 region. Finally, Angius et al. (2018) placed anodal electrodes over the bilateral M1 region and the cathodal electrodes above the ipsilateral shoulders, resulting in an extracephalic tDCS montage that caused increased cortical excitability, reduced perceived exertion, and longer time to fatigue. Together, these recent reports indicate that cephalic and extracephalic montages with the anode over the M1 region are preferable when acute tDCS is applied to extend time to fatigue duration that appears to depend, at least partially, on how perceived exertion is attenuated (Angius et al., 2017).

The inconsistencies found in previous studies might also have been caused, at least in part, by the different modes of exercise adopted. The apparent ergogenicity of tDCS is almost exclusively based on laboratory studies that used single joint isometric exercise or cycling as the mode of exercise (Angius et al., 2017), and thus, it is not known whether these same ergogenic effects are evident in alternative modes of exercise such as running. If improvements in exercise performance are evident following acute tDCS, this would have important implications for performance enhancement in runners. To the best of our knowledge, there are currently no published reports on the effects of tDCS on exercise running performance. The purpose of the present study was therefore to investigate the effects of acute tDCS on performance and related effects on physiological and psychological responses during maximal incremental exercise in recreational runners. We hypothesized that, compared with sham and cathodal tDCS, anodal tDCS applied over the M1 region would (1) significantly attenuate perception of effort, such that a less strenuous perceived exertion would be reported for the same work rate (Okano et al., 2015; Vitor-Costa et al., 2015; Angius et al., 2016); and, therefore, (2) significantly increase exercise performance (i.e., time to exhaustion and peak velocity, Vpeak; Kuipers et al., 1985), regardless of changes in physiological responses (Okano et al., 2015; Vitor-Costa et al., 2015; Angius et al., 2018). We also hypothesized that neither anodal tDCS nor cathodal tDCS would affect affective responses to exercise significantly. Affective states are believed to originate from anatomical brain regions distinct from the primary motor cortex in humans (Oliveri et al., 2003; Van Loon et al., 2010). However, because no study has examined the effects of the brain stimulation with tDCS on affective responses to exercise, this hypothesis is more exploratory in nature.

## Materials and Methods

### Subjects

Thirteen male recreational endurance runners (means ± SD; age 27 ± 5 years, body mass 70 ± 7 kg; height 1.76 ± 0.07 m) were recruited to participate in this study which was approved by the North University of Paraná Ethics Committee. The study was limited to males due to sex differences in motor cortical excitability changes following brain stimulation with tDCS (Kuo et al., 2006). Prior to beginning the tests, a detailed written explanation of the study procedures was provided to all subjects in accordance with the Declaration of Helsinki. None of the participants were smokers or used any dietary supplementation or medication that could affect their somatic and/or cognitive functions. All subjects provided written informed consent and declared to be in good health and none of them presented medical contraindications for participation in the study. All subjects were trained, non-elite recreational endurance runners who were fully familiar with the laboratory testing procedures and habituated to treadmill running. Their training had to consist of 1–2 h endurance running more than four times per week, with a running distance longer than 20 km per week for at least 1 year prior to the start of the study.

### Experimental Procedures

All subjects reported to the laboratory on four separate occasions over a 2-week period that included one preliminary visit and three experimental visits. During the first visit to the laboratory, subjects were familiarized with the testing procedures employed in the study. After the preliminary visit, they returned to the laboratory on three occasions and were randomly assigned using the following site^1^, in a double-blind and counterbalanced fashion, to sham, anodal tDCS, and cathodal tDCS conditions. The subjects were previously instructed to arrive at the laboratory in a rested and fully hydrated state, at least 3 h postprandial, and to refrain from alcohol and caffeine consumption for at least 24-h and 6-h prior to each experimental visit, respectively, to ensure a stable level of motor cortical excitability. They were also asked to abstain from strenuous exercise for at least 24-h prior to each visit to the laboratory. Throughout the study period, subjects were instructed to maintain their normal daily activities and food and fluid intake. All experimental visits were performed at the same time of the day to avoid circadian variance in a temperature-controlled room (range 21–23°C).

Upon arrival at the laboratory, subjects were seated and asked to rest for 10 min before receiving either of the experimental conditions – real tDCS or sham (see next paragraph) – for 20 min. They then completed an incremental ramp exercise test on a motor-driven treadmill (Super ATL, Inbramed, Porto Alegre, Brazil) set at a 1% gradient (Jones and Doust, 1996) to determine the gas exchange threshold (GET) and peak oxygen uptake ($\dot{V}$*O*_2peak_). Following 4 min of baseline, the protocol began with subjects running at 7 km h^-1^ for 6 min, after which the treadmill speed was increased by 1 km h^-1^ every minute until volitional exhaustion (Lansley et al., 2011), despite strong verbal encouragement. The exact time to volitional exhaustion from the onset of the ramp exercise test was recorded to the nearest second (Jones et al., 2004). The speedometer was covered so that subjects, but not evaluators, were blinded to the actual treadmill speed. Breath-by-breath pulmonary gas exchange and ventilation and indices of muscle hemodynamics and oxygenation measured with NIRS data were collected continuously during the ramp exercise test. Ratings of perceived exertion (RPE) and affective valence were assessed at the end of each 2-min stage of the ramp exercise test.

A cephalic tDCS montage similar to the one used by Vitor-Costa et al. (2015) was adopted in the present investigation. This cephalic montage with the anode over the M1 region is preferable when acute tDCS is applied to extend time to fatigue duration in continuous, dynamic, whole-body exercise studies (Angius et al., 2017). Specifically, the electrical current was applied with a portable apparatus consisting of four main components: two rubber electrodes (anode and cathode with an area of 35–36 cm^2^), ammeter (measures the intensity of the electrical current), potentiometer (component that permits manipulation of current intensity), and three batteries (9 V) to generate the current (Vitor-Costa et al., 2015). To ensure good conductance, the electrodes were wrapped in a saline (150 mM NaCl)-soaked sponge current (Vitor-Costa et al., 2015) and elasticated straps were used to secure the electrodes on the scalp (Murphy et al., 2009). The EEG 10–20 international system was used for electrode positioning (Jasper, 1958). The choices of electrode size and placement were intended to induce electrical current in both the left and right sides of the primary motor cortex simultaneously. The center of one electrode (9 × 4 cm) was placed in the Cz region (∼4.5 cm of each side of the primary motor cortex), while the center of the other electrode (7 × 5 cm) was placed on the occipital protuberance (Vitor-Costa et al., 2015). The subjects were stimulated with tDCS (2 mA) for a total duration of 20 min with a 10 s ramp at the beginning and end of stimulation. For the sham condition, the electrodes were placed at the same positions as for the real tDCS. However, stimulation was delivered at 1 mA for 30 s, with a 20 s ramp (Murphy et al., 2009; Vitor-Costa et al., 2015). Using this procedure, subjects are not able to distinguish between sham and real tDCS (12 out of the 13 subjects were naïve to tDCS) (Gandiga et al., 2006).

### Measurements

Pulmonary gas exchange and ventilation were measured breath-by-breath using an automated gas analysis system (K4B^2^, Cosmed, Rome, Italy) (Duffield et al., 2004). A turbine digital transducer measured inspired and expired airflow, and an electrochemical cell O_2_ analyzer and infrared CO_2_ analyzer simultaneously measured expired gases. Subjects wore a tight-fitting facemask that was secured in place by a head cap assembly and attached to the volume transducer. The inspired and expired gas volume and gas concentration signals were continuously sampled via a capillary line connected to the turbine. Prior to each test, the gas analysis system was calibrated using ambient air, in accordance with the manufacturer’s guidelines, against known concentrations of cylinder gases (16% O_2_ and 5% CO_2_) and a precision 3 L calibration syringe (Hans Rudolph, Kansas City, MO, United States). Heart rate (HR) was measured during all tests using short-range radio telemetry (Polar RS800, Polar Electro Oy, Kempele, Finland).

Indices of muscle hemodynamics and oxygenation were assessed using a portable continuous wave NIRS system (Portamon, Artinis Medical System, Elst, Netherlands). The NIRS apparatus emits light at 760- and 850-nm wavelengths from three optodes, with an average optode-detector distance of 35 mm. Penetration depth of the light into tissue was estimated at 17.5 mm, or approximately half the distance between the optode and the detector (Van Beekvelt et al., 2001). The probes of the NIRS apparatus were secured on the right vastus lateralis muscle, ∼10–12 cm above the knee joint and along the vertical axis of the thigh. A surgical marker pen was used to mark probe placement for accurate repositioning throughout the experimental sessions. The probe was covered with black tape to prevent contamination from ambient light. It was then placed in a transparent sealed polyethylene bag for waterproofing and firmly secured to the leg to prevent water leaking into the space between the bag and the skin (Jones et al., 2014). Skinfold thickness at the site of application of the NIRS probe was determined before the first experimental session using a Harpenden skinfold caliper (British Indicators, Burgess Hill, United Kingdom). The calculated value of the combined adipose tissue and skin thickness was less than one-half the distance between the optode and the detector (Ferrari et al., 2004). The NIRS system was connected to a personal computer by Bluetooth for data acquisition (10 Hz), analog-to-digital conversion, and subsequent analysis.

RPE for the overall body was determined by the 6–20 Borg RPE scale (Borg, 1982). This scale is a 15-point single-item measure, ranging from “no exertion at all” (6) to “maximal exertion” (20). Participants were previously anchored to the scale using memory-anchoring procedures (Noble and Robertson, 1996). RPE was assessed at the end of each 2-min stage of the maximal incremental ramp exercise test.

Affective valence was measured using the Feeling Scale (FS) (Hardy and Rejeski, 1989). This scale is an 11-point single-item measure, ranging from “very bad” (-5) to “very good” (+5). In a counterbalanced manner, 6–20 Borg RPE and FS scales were administered at the end of each 2-min stage of the maximal incremental exercise test. Participants rated “how” and “what” they felt at these moments (DaSilva et al., 2011; Vandoni et al., 2016). Standard definitions of perceived exertion and affective valence, along with separate instructional sets for the 6–20 Borg RPE and FS scales, were read to the participants before the tests (Ekkekakis et al., 2005; Noble and Robertson, 1996). During the maximal incremental ramp exercise test, the 6–20 Borg RPE and FS were in full view of the participants at all times.

### Data Analysis

The breath-by-breath pulmonary gas exchange and ventilation data from each test were examined to exclude occasional errant breaths by removing values that lay outside 4 SD from the local mean determined using a 5-breath rolling average (Lamarra et al., 1987). Filtered pulmonary gas exchange and ventilation data were subsequently linearly interpolated to provide second-by-second values and averaged over consecutive 10 s periods. The baseline $\dot{V}$O_2_ ($\dot{V}$O_2baseline_) and pulmonary ventilation ($\dot{V}$_E_) at baseline ($\dot{V}$_E__baseline_) were defined as the mean values measured over the 90 s baseline period. The end-exercise $\dot{V}$O_2_ ($\dot{V}$O_2__peak_) and $\dot{V}$_E_ ($\dot{V}$_Emax_) were defined as the mean values measured over the final 30 s of exercise (Wylie et al., 2013). The GET, an index of anaerobic threshold, was determined from data averaged in 10-s time bins by plotting the ventilatory equivalent ($\dot{V}$_E_ /$\dot{V}$O_2_) as a function of $\dot{V}$O_2_ to detect the point during exercise where this curve reached its lowest value (Hagan and Smith, 1984; Hollmann, 2001). Peak HR (HR_peak_) was defined as the peak value measured over the final 30 s of exercise.

The second-by-second NIRS data from each test were averaged over consecutive 10 s periods and normalized to the total amplitude of the response. The baseline NIRS data were calculated for oxyhemoglobin [HbO_2_] and deoxyhemoglobin [HHb] concentrations and tissue oxygenation index (TOI), based on the principles of the Beer-Lambert law (Van Beekvelt et al., 2001), and were defined as the mean values measured over the final 60 s of the baseline period (set as 0%). The end-exercise NIRS data were defined as the mean values measured over the final 30 s of exercise and set as 100% (Boone et al., 2009).

### Statistical Analysis

Unless stated otherwise, data are presented as mean (±SD). The distribution of the data was checked by the Shapiro–Wilk test, and the results showed a normal Gaussian distribution. Mauchly’s test of sphericity was used to test this assumption, and a Greenhouse–Geisser procedure was used when necessary. Repeated-measures analysis of variance followed by the Bonferroni *post hoc* test were used for comparison of time to exhaustion and V_peak_ between the different brain stimulation conditions (sham, anodal tDCS, and cathodal tDCS). Two-way repeated-measures analysis of variance were used for all other comparisons, using the brain stimulation conditions and time (*baseline, min 0* (i.e., end of warm-up), *min 2, min 4, min 6, min 8*, and *end test* for exercise test variables) as main factors. When a significant simple main effect of brain stimulation condition or time was found, Bonferroni follow-up test was performed. Effect sizes were calculated using partial eta squared ($\eta_{p}^{2}$) and thresholds for small, moderate, and large effect sizes were set at 0.01, 0.08, and >0.13, respectively (Cohen, 1988). Statistical analyses were performed using SPSS statistical software (version 24.0 for Windows; SPSS Inc., Chicago, IL, United States), with *a priori* statistical significance set at *P* < 0.05.

Sample size was estimated (G^∗^Power software, version 03.1.9.2; Faul et al., 2007) on the basis of a recent study by Vitor-Costa et al. (2015) that reported a significant improvement in time to exhaustion during cycling exercise following anodal tDCS applied over the M1 region via a cephalic montage. It was estimated that a sample size of 9 was required to achieve a statistical power of 80% at an alpha level of 0.05 with a moderate Cohen’s *d* effect size of 0.60 (Cohen, 1988).

## Results

All subjects completed the three experimental visits. Participants reported similar habitual physical activity patterns and dietary intake in the 24-h prior to each visit to the laboratory. Subjects experienced an itching sensation under the electrodes during all the tDCS conditions, however, the brain stimulation regimen was well-tolerated and no harmful side effects were reported.

**FIGURE 1:**
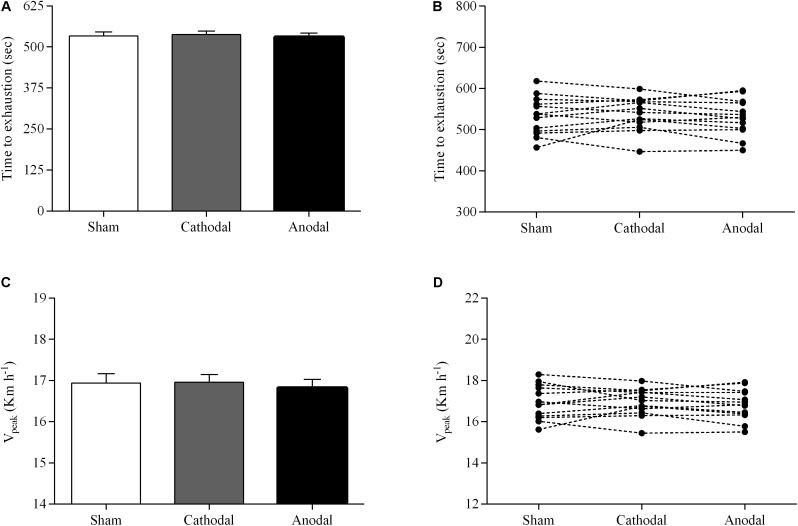
Group mean **(A,C)** and individual **(B,D)** responses of time to exhaustion and V_peak_ during a ramp incremental exercise until volitional exhaustion, following sham (white bar), cathodal tDCS (gray bar), and anodal tDCS (black bar). Data are shown as mean (±SEM).

The parameters of exercise performance for the three brain stimulation conditions are shown in **Figure 1**. There was no significant effect of brain stimulation condition on time to exhaustion (*P* > 0.05; $\eta_{p}^{2}$ = 0.05). The ramp exercise protocol lasted, on average, 537 ± 40 s and 530 ± 44 s in both cathodal tDCS and anodal tDCS conditions, respectively, whereas the ramp exercise protocol lasted 533 ± 46 s in sham condition (**Figure 1A**). No significant effect of brain stimulation condition was also found for V_peak_ (*P* > 0.05; $\eta_{p}^{2}$ = 0.10). Specifically, subjects reached a similar V_peak_ in both cathodal tDCS and anodal tDCS conditions (16.9 ± 0.6 km h^-1^ and 16.8 ± 0.7 km h^-1^) compared with sham condition (16.9 ± 0.8 km h^-1^) (**Figure 1C**).

**FIGURE 2:**
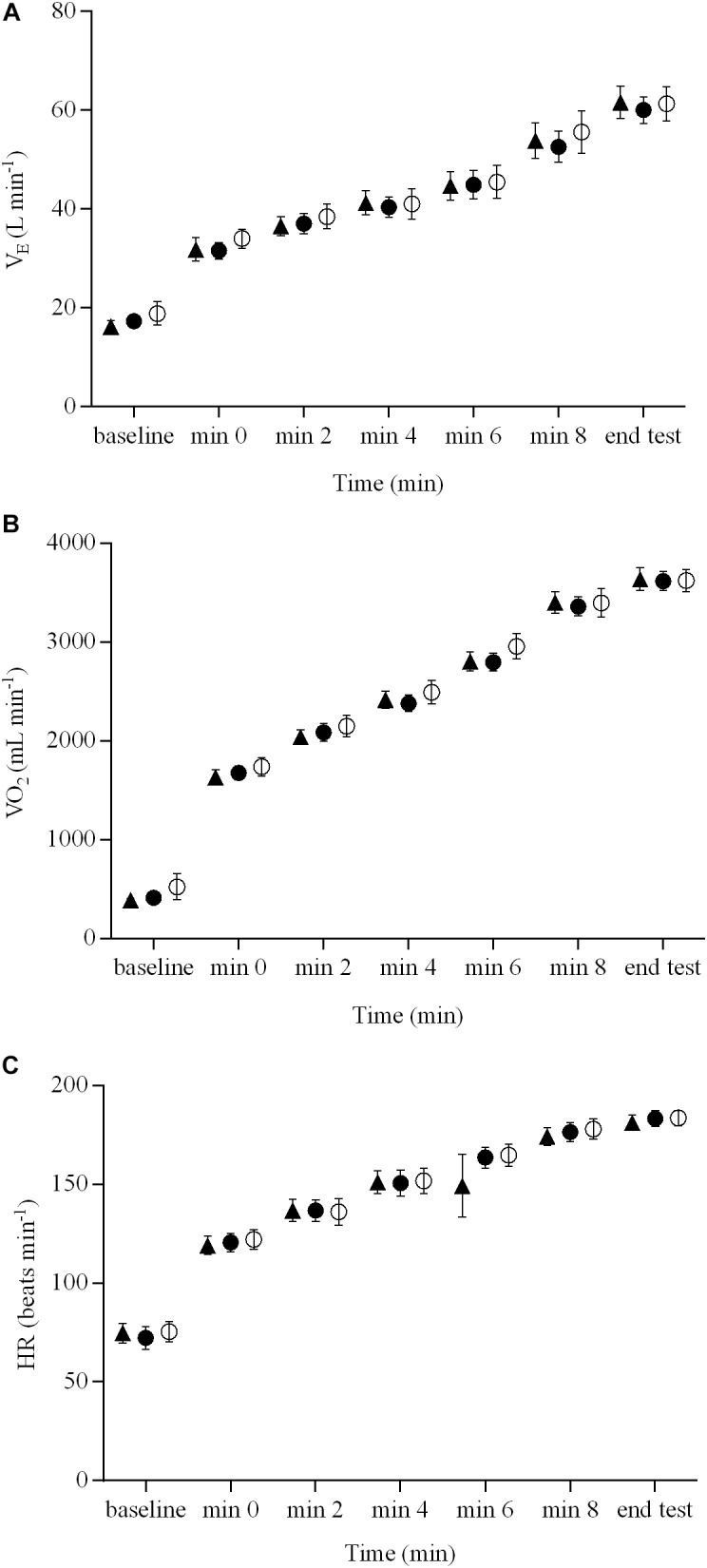
Pulmonary gas exchange and cardiovascular responses during a ramp incremental exercise until volitional exhaustion, following sham, cathodal tDCS, and anodal tDCS. Responses following sham are represented as open circles (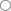), while the cathodal tDCS and anodal tDCS responses are shown as solid circles (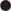) and triangles (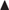), respectively. Data are presented as mean (±SEM). **(A)** Group mean $\dot{V}$_E_ response to ramp incremental exercise. **(B)** Group mean $\dot{V}$O_2_ response to ramp incremental exercise. **(C)** Group mean HR response to ramp incremental exercise.

The pulmonary gas exchange and cardiovascular responses for the three brain stimulation conditions are presented in **Figure 2**. There were no brain stimulation condition effects (range $\eta_{p}^{2}$ = 0.05 – 0.11) or brain stimulation condition × time interactions (range $\eta_{p}^{2}$ = 0.08 – 0.10) for $\dot{V}$_E_, $\dot{V}$O_2_, or HR responses during the ramp exercise test (*P* > 0.05). However, there were significant main effects of time (range $\eta_{p}^{2}$ = 0.82 – 0.96) for all of these variables (*P* < 0.01). $\dot{V}$O_2peak_ did not differ significantly between the three brain stimulation conditions (3621 ± 394, 3613 ± 357, and 3636 ± 425 mL min^-1^ for sham, cathodal tDCS, and anodal tDCS conditions, respectively; *P* > 0.05). The GET, determined during the ramp exercise test, occurred, on average, at the same percentage of $\dot{V}$O_2peak_ for both sham tDCS (52 ± 4%) and real tDCS (cathodal, 52 ± 9%, and anodal, 53 ± 1%), respectively (*P* > 0.05).

**FIGURE 3:**
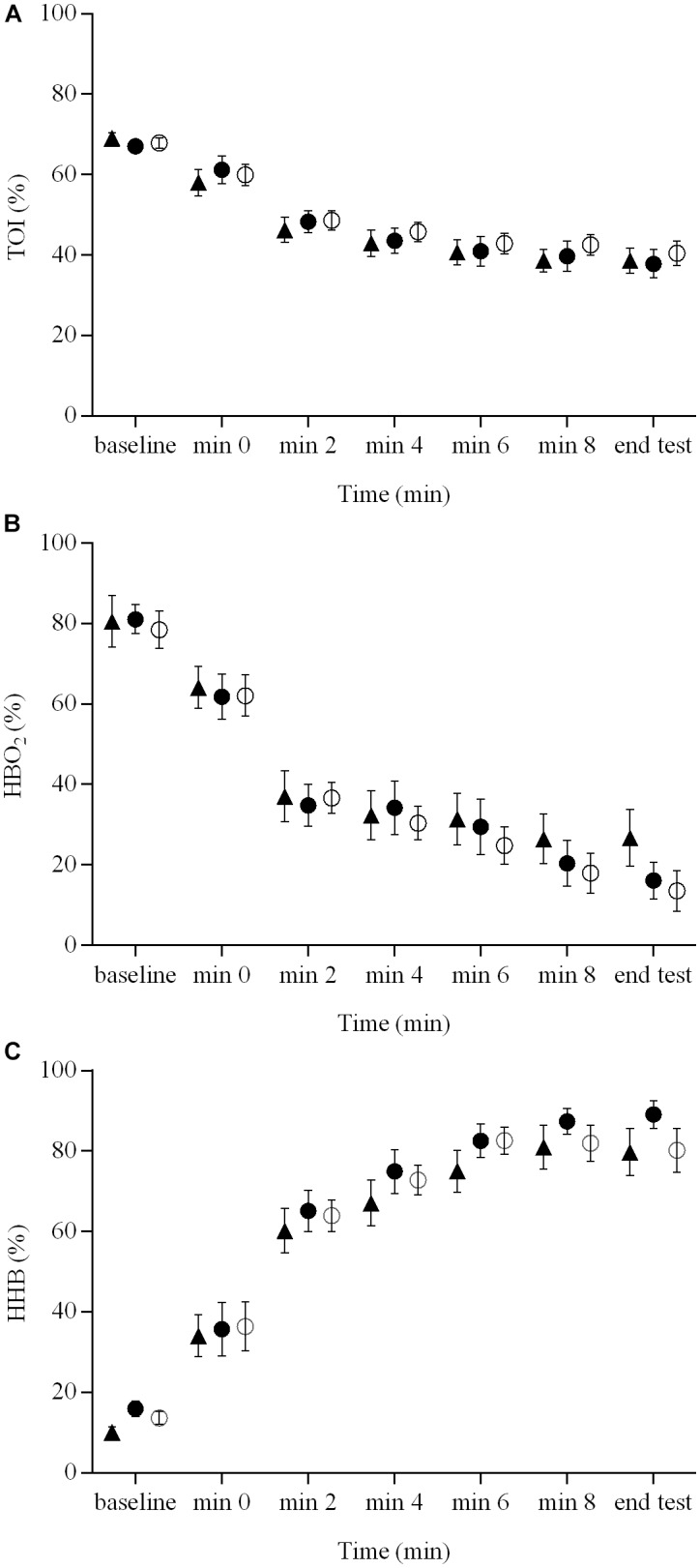
Indices of muscle hemodynamics and oxygenation measured with NIRS during a ramp incremental exercise until volitional exhaustion, following sham, cathodal tDCS, and anodal tDCS. Responses following sham are represented as open circles (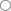), while the cathodal tDCS and anodal tDCS responses are shown as solid circles (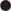) and triangles (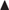), respectively. Data are presented as mean (±SEM). **(A)** Group mean TOI response to ramp incremental exercise. **(B)** Group mean HbO_2_ response to ramp incremental exercise. **(C)** Group mean HHb response to ramp incremental exercise.

The indices of muscle hemodynamics and oxygenation measured with NIRS are illustrated in **Figure 3**. There were no brain stimulation condition effects (range $\eta_{p}^{2}$ = 0.05 – 0.10) or brain stimulation condition × time interactions (range $\eta_{p}^{2}$ = 0.03 – 0.11) for the NIRS-derived [HbO_2_], [HHb], or TOI responses during the ramp exercise test (*P* > 0.05). However, there were significant main effects of time (range $\eta_{p}^{2}$ = 0.83 – 0.87) for all of these variables (*P* < 0.01).

**FIGURE 4:**
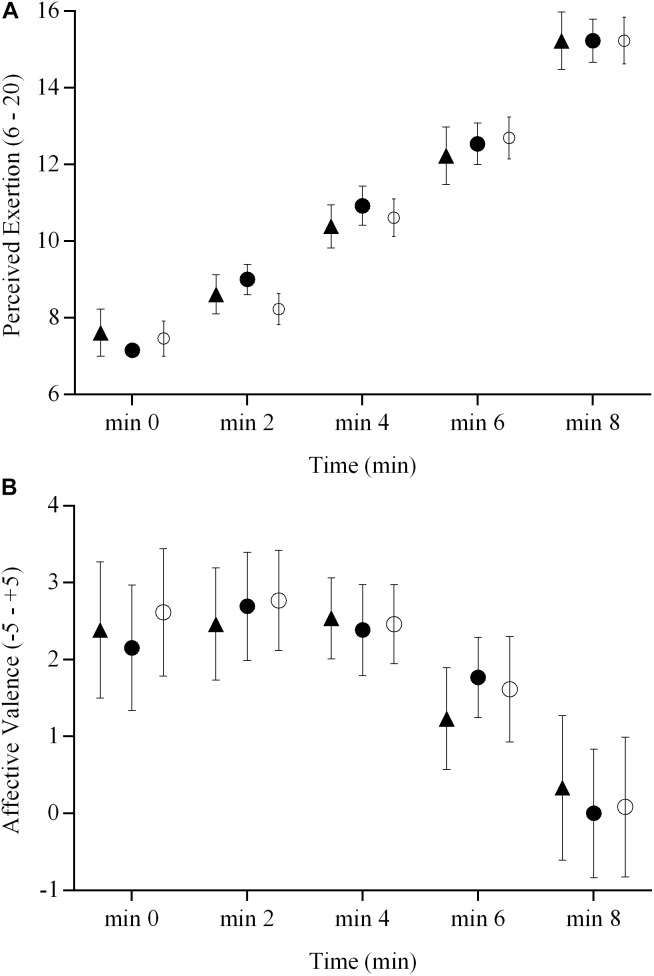
RPE and affective valence responses during a ramp incremental exercise until volitional exhaustion, following sham, cathodal tDCS, and anodal tDCS. Responses following sham are represented as open circles (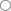), while the cathodal tDCS and anodal tDCS responses are shown as solid circles (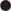) and triangles (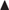), respectively. Data are presented as mean (±SEM). **(A)** Group mean RPE response to ramp incremental exercise. **(B)** Group mean affective valence response to ramp incremental exercise.

The RPE and affective valence responses for the three brain stimulation conditions are presented in **Figure 4**. There were no brain stimulation condition effects ($\eta_{p}^{2}$ = 0.06 and 0.08) or brain stimulation condition × time interactions ($\eta_{p}^{2}$ = 0.08 and 0.09) on the RPE and affective valence responses during the ramp exercise test (*P* > 0.05), respectively. However, there were significant main effects of time ($\eta_{p}^{2}$ = 0.92 and 0.28, respectively for RPE and affective valence) for both variables (*P* < 0.05).

## Discussion

To the best of our knowledge, this study is the first to characterize the effects of brain stimulation applied over the M1 region via a cephalic montage on exercise performance and psychophysiological responses to incremental large muscle group exercise in moderately trained runners. Specifically, we studied how acute tDCS of three different polarities impact perceived exertion, affective valence, exercise tolerance and associated physiological responses to ramp exercise. The principal novel finding of this investigation was that, compared with sham, neither anodal tDCS nor cathodal tDCS altered the tolerable duration of exercise in recreational endurance runners. This is somewhat surprising considering the recent reports that anodal tDCS improved isometric time to task failure tests of isolated muscle groups (Williams et al., 2013; Abdelmoula et al., 2016; Angius et al., 2016) and enhanced exercise performance in cycling studies (Angius et al., 2015; Okano et al., 2015; Vitor-Costa et al., 2015; Barwood et al., 2016). It is noteworthy, however, that this lack of improvements of exercise tolerance occurred without effects on the perceived exertion or on the affective valence.

In contrast with some (Okano et al., 2015; Vitor-Costa et al., 2015; Angius et al., 2018) but not all (Angius et al., 2015; Barwood et al., 2016) previous studies, anodal tDCS did not significantly alter exercise performance. Previous studies have typically reported improvements in various parameters of endurance performance of ∼4–23% following brain stimulation (Okano et al., 2015; Vitor-Costa et al., 2015; Angius et al., 2018). In the pioneer cycling study of Okano et al. (2015), significant changes in peak power output were observed when a cephalic montage was administered with a single anodal electrode over T3 of the insular cortex and with the cathode over the contralateral supraorbital area, fp2. Similarly, Vitor-Costa et al. (2015) reported a significant improvement in time to exhaustion during cycling exercise following anodal tDCS applied over the M1 region via a cephalic montage. This finding provided evidence that anodal stimulation of the cortical area upstream of M1 (primary motor cortex) may improve cycling endurance performance. Alternatively, or additionally, Angius et al. (2018) placed anodal electrodes over the bilateral M1 region and the cathodal electrodes above the ipsilateral shoulders, resulting in an extracephalic tDCS montage that caused increased cortical excitability and longer time to fatigue. The authors therefore suggested that an extracephalic tDCS montage may be preferable for continuous, dynamic, whole-body exercise.

Differently from Angius et al. (2018), the present investigation adopted a cephalic tDCS montage similar to the one used by Vitor-Costa et al. (2015). Consistent with previous studies (Angius et al., 2015, 2018; Vitor-Costa et al., 2015; Barwood et al., 2016), there were slight but non-significant changes in physiological responses to exercise, conflating to produce a non-significant improvement in exercise tolerance in moderately trained runners. The apparent lack of any acute ergogenic effect from anodal tDCS found in the current study may be attributed, in part, to the electrode montage. It appears that a bilateral extracephalic montage is more appropriate when tDCS is applied to enhance exercise performance. Cephalic tDCS montages may typically cause effects under the cathode that may modulate or even negate the effect of the anode over M1 (Angius et al., 2015, 2016). Otherwise, extracephalic tDCS montages simultaneously stimulate both primary motor cortices while also avoiding the negative effects of the cathode over other brain areas (Vandermeeren et al., 2010; Angius et al., 2018). Furthermore, the tDCS montage adopted in the current study placed one electrode over the occipital protuberance, and potentially, the direction of current between the two electrodes could also have interfered with other brain areas (Lang et al., 2005; Polania et al., 2011), thus reflecting in both physiological and psychological responses to exercise and running performance. Therefore, future studies with different tDCS characteristics in terms of electrode montage and location are necessary to further examine the relationship between brain stimulation and endurance performance in this population.

The absence of any significant effect of anodal tDCS on exercise performance in the present study may also be due to a failure of brain stimulation to reduce RPE. Earlier reports indicated that anodal tDCS extended time to fatigue duration in parallel with a lower RPE (Williams et al., 2013; Okano et al., 2015; Vitor-Costa et al., 2015; Angius et al., 2016). Theoretically, tDCS administration may improve exercise tolerance by lessening the discomfort levels and consequently decreasing the RPE (Angius et al., 2017). These changes in RPE have been associated with the activity of various areas of the motor cortex, including the premotor and primary motor areas (de Morree et al., 2012, 2014). Thus, when increasing the excitability of the motor cortex by anodal tDCS administration, less excitatory input into the stimulated cortical area is required to produce a given force or power, and hence, a lower RPE would be expected (Goodall et al., 2013; Takarada et al., 2014). Whether the absence of any significant effect of anodal tDCS on RPE observed in the present study might be attributed to increased excitability of the motor cortex and reduced neural drive necessary to perform the task remains to be elucidated. However, tDCS administration with larger electrodes might also have stimulated adjacent cortical areas by influencing the sensorimotor integration during muscular contraction without affecting motor command (Abdelmoula et al., 2016). This hypothesis, however, could also not be tested in the context of the current study design.

To our knowledge, this is the first study to investigate the effects of brain stimulation on affective responses to exercise. Affective states arise from two diverse neurophysiological systems, one related to valence (a pleasure – displeasure continuum) and the other to arousal, or alertness (Russell, 1980). In the present study, emphasis was placed on affective valence as this dimension is of relevance for the analysis of (un)pleasant exercise feelings and future exercise participation (Ekkekakis et al., 2005; Williams et al., 2008; DaSilva et al., 2011). Similar to RPE, affective responses during the ramp exercise test did not differ between the three experimental conditions at any time. The interpretation of this finding is not straightforward, however. The anodal tDCS montage adopted in the current study placed one electrode over the Cz region, a brain area not usually associated with the regulation of (un)pleasant feelings (Oliveri et al., 2003; Van Loon et al., 2010). In fact, affective states appear to originate from a complex myriad of anatomical brain regions, such as the prefrontal cortex, including portions of orbitofrontal, insula, and anterior cingulate cortices, as well as subcortical limbic structures such as nucleus accumbens, ventral pallidum, and amygdala (Berridge and Kringelbach, 2015). It should be noted, however, that tDCS stimulates not only the cortical area directly under the electrodes, but also subcortical structures, since there are connections within the cortico-cortical neural networks (Lang et al., 2005; Polania et al., 2011). Therefore, the apparent lack of any acute effect from tDCS administration on psychophysiological responses to exercise found in the current study may be attributed, in part, to the electrode montage and location as well as the magnitude of the cortical – and subcortical – excitability. This suggestion is naturally speculative and awaits further investigation.

It should be noted that recent studies indicate sex differences, possibly due to the regulatory effects of hormones involved in the menstrual cycle in women (Smith et al., 1999, 2002), in the modulation of human cortical plasticity (Kuo et al., 2006; Chaieb et al., 2008; Russell et al., 2017). Compared to men, women are more susceptible to motor cortical excitability changes following brain stimulation with tDCS (Kuo et al., 2006). Therefore, it is not clear whether the results of the present study can be applied to female recreational endurance runners. The sex differences in endurance performance following brain stimulation are presently not known and are likely to be a fertile area for further research.

## Conclusion

This study provides the first description of the effects of brain stimulation applied over the M1 region via a cephalic montage on exercise performance and psychophysiological responses to exercise in recreational endurance runners. Our results suggest that this simple non-invasive neuromodulatory technique would not be effective to alter physiological responses, perception of effort, pleasant feelings, or exercise tolerance in runners during activities requiring large muscle mass. These findings have important implications for the use of the brain stimulation to enhance endurance performance in recreationally trained runners. Whether differences in electrode tDCS montage and location, as well as current intensities or stimulation durations, would produce dissimilar effects on running performance cannot be concluded, and should be topic of future studies.

## Author Contributions

CFB, MV-C, and LG developed the concept and designed the research. JG, AB, and MV-C performed the experiments. CFB, JG, AB, and LA analyzed the data. CB, CFB, JG, AB, MV-C, LA, and LG interpreted the results of the experiments. CFB, JG, AB, and MV-C prepared the figures. CB, CFB, MV-C, and LG drafted the manuscript. CB, CFB, MV-C, and LG edited and revised the manuscript. CB, CFB, JG, AB, MV-C, LA, and LG approved the final version of the manuscript.

## Conflict of Interest Statement

The authors declare that the research was conducted in the absence of any commercial or financial relationships that could be construed as a potential conflict of interest.

## Footnotes

1 http://www.randomization.com
